# Characterizing ADRs of Enfortumab vedotin and Erdafitinib in bladder cancer treatment: a descriptive analysis from WHO-VigiAccess

**DOI:** 10.3389/fphar.2024.1503154

**Published:** 2024-12-06

**Authors:** Yuanbin Huang, Meiqi Xu, Xinmiao Ma, Wei Wang, Chen Shen, Fei Liu, Zhiqi Chen, Jiawen Wang, Qian Guo, Xiancheng Li

**Affiliations:** ^1^ Second Affiliated Hospital of Dalian Medical University, Dalian Medical University, Dalian, China; ^2^ Department of Urology, Fujian Provincial Hospital, Fuzhou, China; ^3^ Department of Rhinology, The First Affiliated Hospital of Zhengzhou University, Zhengzhou University, Zhengzhou, China

**Keywords:** adverse drug reaction, Enfortumab vedotin, Erdafitinib, pharmacovigilance, spontaneous reporting, WHO-VigiAccess database

## Abstract

**Introduction:**

Enfortumab vedotin (EV) and Erdafitinib are effective therapeutic drugs for bladder cancer patients following post-chemotherapy and immunotherapy. This study assessed adverse drug reactions (ADRs) from both drugs, comparing their safety profiles to guide clinical use.

**Methods:**

A retrospective descriptive analysis was conducted on ADR reports for EV and Erdafitinib from the World Health Organization (WHO)-VigiAccess database. Data on patient demographics, system organ classes (SOCs), global patient regions, symptoms, and ADRs frequencies were analyzed and compared.

**Results:**

As of 2024, 3,438 ADR reports were identified (2,257 for EV and 1,181 for Erdafitinib). The number of adverse reaction reports for EV is significantly higher than that for Erdafitinib. Among them, the SOC with the most adverse signals is gastrointestinal disorders, with the top five reports being nausea, gastrointestinal disorders, dry mouth, abdominal pain, and diarrhea. The top five reported adverse events (AEs) for EV are as follows: skin and subcutaneous tissue disorders (20.70%), general disorders and administration site conditions (14.23%), nervous system disorders (11.12%), gastrointestinal disorders (7.78%), and metabolism and nutrition disorders (6.47%). In contrast, the top five AEs for Erdafitinib are: general disorders and administration site conditions (25.36%), skin and subcutaneous tissue disorders (10.94%), gastrointestinal disorders (10.19%), eye disorders (9.21%), and injury poisoning and procedural complications (7.31%).

**Conclusion:**

Our study identified and compared potential and novel ADRs between EV and Erdafitinib, providing key insights into their safety profiles and highlighting the need for personalized treatment strategies based on individual patient risk factors.

## Introduction

Bladder cancer is one of the most prevalent cancers worldwide, accounting for approximately 3.0% of all cancer cases and 2.1% of cancer-related deaths globally (Compérat et al., 2022). While early-stage bladder cancer typically responds well to treatment, the prognosis for advanced or metastatic disease remains poor, with a 5-year survival rate of only 5%–7%. First-line treatments for advanced bladder cancer generally involve cisplatin- or carboplatin-based chemotherapy, which often yields suboptimal results. Despite initial responses, many patients experience relapse, with overall survival usually less than 9 months (Galsky et al., 2012). This underscores the urgent need for novel therapeutic strategies to enhance outcomes for patients with advanced bladder cancer. Enfortumab vedotin (EV) is an antibody-drug conjugate (ADC) that targets nectin-4, a protein expressed on the surface of advanced bladder cancer cells. It has received approval from both the Food and Drug Administration (FDA) and the European Medicines Agency (EMA) for the treatment of adults with advanced bladder cancer who have previously undergone platinum-based chemotherapy and PD(L)-1 inhibitors (Talukder et al., 2023). Furthermore, EV is FDA-approved for patients who are ineligible for cisplatin, regardless of prior treatments, and can also be combined with pembrolizumab (Hoffman-Censits and Maldonado, 2022). The safety and efficacy of EV were validated in the phase III open-label, randomized, multicenter EV-301 study, which included patients with advanced bladder cancer who had previously been treated with platinum-based chemotherapy and PD(L)-1 inhibitors. Compared to chemotherapy (e.g., docetaxel, paclitaxel, or vinflunine), EV significantly prolonged overall survival (OS, HR = 0.70, 95% CI: 0.56–0.89) and progression-free survival (PFS, HR = 0.62, 95% CI: 0.51–0.75) after a median follow-up of 11.1 months (Powles et al., 2021a). A survey indicated that the incidence of adverse events (AEs) in the EV group was comparable to that in the chemotherapy group, recorded at 93.9% and 91.8%, respectively. Furthermore, the incidence of grade ≥3 AEs was 51.4% in the EV group, compared to 49.8% in the chemotherapy group (Powles et al., 2021b). Among the EV group, grade ≥3 serious AEs occurring in more than 5% of patients included maculopapular rash (7.4%), fatigue (6.4%), and neutropenia (6.1%). Conversely, the chemotherapy group exhibited higher incidences of neutropenia (13.4%), anemia (7.6%), leukopenia (6.9%), neutropenic fever (6.2%), and febrile neutropenia (5.5%) (Rosenberg et al., 2023).

Erdafitinib is an oral fibroblast growth factor receptor (FGFR) kinase inhibitor that has been approved by the FDA for the treatment of adults with advanced bladder cancer harboring susceptible FGFR3 genetic alterations, particularly in patients who have progressed after at least one line of systemic therapy, including PD-1 or PD-L1 inhibitors (Ascione et al., 2023). In a phase III randomized controlled trial, Erdafitinib significantly improved overall survival (OS) (HR 0.64; 95% CI: 0.47–0.88) and progression-free survival (PFS) (HR 0.58; 95% CI: 0.44–0.78) compared to chemotherapy (e.g., docetaxel or vinflunine), demonstrating superior clinical efficacy (Franza et al., 2022). Furthermore, Erdafitinib’s established safety profile includes grade 3 or 4 AEs, such as dermatologic conditions (11.9%), nail disorders (11.1%), central serous chorioretinopathy (2.2%), and other ocular diseases (2.2%) (Loriot et al., 2023).

Both EV and Erdafitinib have demonstrated promising efficacy in the treatment of advanced bladder cancer. A critical aspect of their clinical application is the understanding of their distinct safety profiles. Recent studies have identified common adverse drug reactions (ADRs) associated with each treatment. EV is frequently linked to peripheral neuropathy, skin reactions, fatigue, and neutropenia (Hanna, 2019), whereas ADRs of Erdafitinib are primarily related to its inhibition of FGFR, manifesting as dermatologic and nail disorders, central serous chorioretinopathy, and hyperphosphatemia (Van Sanden et al., 2024). A narrative review has highlighted the rapid advancements in genitourinary cancer treatments, emphasizing the significant progress in therapeutic options alongside the concurrent challenges posed by the adverse effects of commonly used drugs, including EV and Erdafitinib. Among these challenges, dermatologic toxicity, encompassing changes to the skin, nails, and hair, emerges as one of the most prevalent side effects. Notably, the incidence and types of skin-related toxicities differ between EV and Erdafitinib. EV is often associated with severe skin reactions, such as maculopapular rash and toxic epidermal necrolysis, whereas Erdafitinib more commonly causes dermatologic and nail disorders, as well as central serous chorioretinopathy (Daher et al., 2024). Furthermore, a matching-adjusted indirect treatment comparison has revealed that while EV and Erdafitinib demonstrate comparable efficacy in overall survival and progression-free survival, Erdafitinib appears to provide a higher probability of achieving a deeper response. However, this benefit comes with a slightly higher incidence of ADRs compared to EV, although the severity of these events is generally low (Van Sanden et al., 2024). These findings underscore the importance of comparing the safety profiles of EV and Erdafitinib, as understanding the specific ADRs associated with each drug is crucial for optimizing treatment strategies.

Spontaneous Reporting Systems (SRS) provide invaluable real-world safety data on drugs and vaccines, enabling the early detection of previously unrecognized ADRs, facilitating treatment comparisons, and offering insights into ADRs mechanisms (Noguchi et al., 2019). The World Health Organization (WHO)-VigiAccess, launched by the WHO in 2015, grants public access to VigiBase, a global repository of reported drug side effects. By analyzing data from this database, researchers can identify new associations between drugs and AEs and further confirm existing clinical links (Zhou et al., 2024). This study uniquely leverages the WHO-VigiAccess database to compare the incidence of ADRs between EV and Erdafitinib, with a focus on differences in reporting rates between the two treatments. By highlighting these novel findings, we aim to enhance clinical decision-making, enabling better personalized treatment strategies that optimize both efficacy and quality of life for patients with advanced bladder cancer.

## Materials and methods

### Drug sample

Table 1 provides an overview of the clinical studies we have conducted on EV and Erdafitinib for the treatment of bladder cancer (National Center for Biotechnology Information, 2024a; National Center for Biotechnology Information, 2024b). Following the binding of the antibody component of EV to nectin-4, EV undergoes internalization, during which the cleavable linker is cleaved by lysosomal proteases, resulting in the release of monomethyl auristatin E (MMAE) (Challita-Eid et al., 2016). Erdafitinib, a kinase inhibitor, binds to and inhibits FGFR phosphorylation and signaling, thereby reducing cell viability in cell lines exhibiting FGFR genetic alterations, including point mutations, amplifications, and fusions. As the first targeted therapy for metastatic bladder cancer, Erdafitinib demonstrates significant therapeutic potential and value (Guercio et al., 2023).

**TABLE 1:** Overview of Enfortumab vedotin and Erdafitinib.

Drug name	Chemical name	Structure	Main treatment	First marketed year
Enfortumab vedotin	Anti-Nectin 4 ADC ASG-22CE	C_6468_H_10012_N_1720_O_2038_S_44_	Advanced/metastatic urothelial carcinoma, particularly in patients previously treated with platinum-based chemotherapy and PD-1/PD-L1 inhibitors	2019
Erdafitinib	N1-(3,5-dimethoxyphenyl)-N2-isopropyl-N1-(3-(1-methyl-1H-pyrazol-4-yl)quinoxalin-6-yl)ethane-1,2-diamine	C_25_H_30_N_6_O_2_	Advanced/metastatic urothelial carcinoma with FGFR3 or FGFR2 genetic alterations	2019

Currently, both novel drugs have shown promising efficacy in the treatment of bladder cancer; however, due to the short time on the market, reports on the ADRs associated with these medications remain incomplete.

### Data sources

On 29 August 2024, ADRs associated with EV and Erdafitinib were searched under their common names in WHO-VigiAccess, which provides public access to global drug safety data, aimed at enhancing the transparency of ADR reporting. This platform enables users to view and analyze safety data reported by the global pharmacovigilance network, assisting the public, researchers, and healthcare professionals in understanding drug safety risks and making more informed clinical decisions. The database is accessible via an online platform, offering seamless access to ADR monitoring, with the login URL being https://www.vigiaccess.org. We collected data on gender, age group, year, system organ classes, and symptoms associated with the recorded ADRs from the annual ADR reports of disease data. Descriptive statistics for this study were computed using Excel 2019. This study objectively analyzed the ADRs of the two drugs based on the retrieved data, focusing on the differences in the incidence of ADRs between the two drugs in System Organ Classes (SOCs), the primary symptoms of ADRs (selecting the top twenty symptoms with the highest incidence), and the specific symptom incidence of ADRs in SOCs, as well as the differences in the specific symptom incidence of ADRs between the two drugs in SOCs. The SOC and preferred terms (PTs) are based on the Medical Dictionary of Regulatory Activities (MedDRA). Consequently, we examined the records of the two drugs used for bladder cancer treatment and identified all individual AEs to delineate the toxicity spectrum based on the recorded MedDRA SOCs and PT levels. The reporting terms utilized in MedDRA are derived from several dictionaries, including the World Health Organization Adverse Reaction Terminology (WHO-ART). The SOC categorizes 27 items and selects 20 items directly related to disease symptoms for analysis. In this study, we will emphasize PTs, which represent to the level of publicly accessible information in the VigiBase database through WHO-VigiAccess.

To analyze the results of the detected safety signals, we employed result codes to classify them into three severe categories: death, hospitalization, and major events (including life-threatening events, disabilities, and congenital abnormalities) (Li et al., 2023a; Li et al., 2023b).

### Statistical analysis

This study employs a retrospective quantitative research design. Utilizing Excel for descriptive analysis, we examine the characteristics of victims of ADRs associated with EV and Erdafitinib. The ADR reporting rate for each drug is defined as the number of reported ADR symptoms divided by the total number of ADR reports. Common adverse reactions for various drugs are identified as the top 20 symptoms with the highest ADR reporting rates. We calculate the incidence of reported adverse reaction symptoms for each drug and conduct a descriptive comparative analysis. Descriptive variables are classified using frequency and percentage (Yang et al., 2024).

## Result

### Description of study cases

Table 2 shows the characteristics of ADR reports for EV and Erdafitinib. Reports of adverse reactions for EV and Erdafitinib were first recorded in the WHO-VigiAccess database in 2018. As of 2024, the WHO has documented a total of 3,438 reports for both drugs, with EV accounting for 2,257 reports and Erdafitinib for 1,181 reports. The number of adverse reaction reports for EV is significantly higher than that for Erdafitinib.

**TABLE 2:** Characteristics of ADR reports of Enfortumab vedotin and Erdafitinib.

	Enfortumab vedotin	Erdafitinib
Number of ADR reports	2,257	1181
Female	526 (23.3%)	333 (28.2%)
Male	1665 (73.8%)	548 (46.4%)
Unknown	66 (2.9%)	300 (25.4%)
<18	0 (0%)	20 (1.7%)
18–44	25 (1.1%)	32 (2.7%)
45–64	353 (15.6%)	125 (10.6%)
65–74	574 (25.4%)	155 (13.1%)
≥75	457 (20.2%)	120 (10.2%)
Unknown	848 (37.6%)	729 (61.7%)
Africa	1 (0.1%)	33 (2.8%)
Americas	1154 (51.1%)	698 (59.1%)
Asia	70 (3.1%)	38 (3.2%)
Europe	1007 (44.6%)	409 (34.6%)
Oceania	25 (1.1%)	3 (0.3%)
2024	624 (27.6%)	180 (15.2%)
2023	709 (31.4%)	287 (24.3%)
2022	423 (18.7%)	267 (22.6%)
2021	310 (13.7%)	230 (19.5%)
2020	289 (8.4%)	176 (14.9%)
2019	1 (0.1%)	39 (3.3%)
2018	1 (0.1%)	2 (0.2%)

Among the 3,438 reports involving these two drugs, excluding those with unknown gender, the number of adverse reaction reports for males is notably higher than for females, particularly for EV (males 73.8%, females 23.3%). The gender distribution for Erdafitinib is relatively more balanced (males 46.4%, females 28.2%). Importantly, a substantial proportion of Erdafitinib reports (25.4%) have an unknown gender. After excluding reports with unknown age, adverse reaction reports for EV are primarily concentrated in the age groups of 65–74 years (25.4%) and ≥75 years (20.2%). In contrast, reports for Erdafitinib are mainly concentrated in the 65–74 years age group (13.1%), although a majority of reports (61.7%) have an unknown age. Geographically, reports of AEs for both drugs predominantly originate from the Americas (EV 51.1%, Erdafitinib 59.1%) and Europe (EV 44.6%, Erdafitinib 34.6%). With a relatively small proportion of reports from Asia, Africa, and Oceania. Table 2 also illustrates the reporting years for each drug. Reports of adverse reactions for both drugs peaked in 2023 (EV at 31.4%, Erdafitinib at 24.3%), with anotable increase in reporting observed since 2019. Notably, EV exhibited a particularly significant surge in 2023.

### Distribution of EV and Erdafitinib in 20 SOCs

Table 3 presents the reporting rates for 20 SOCs with EV and Erdafitinib. The incidence of skin and subcutaneous tissue disorders, as well as nervous system disorders, related to EV is significantly higher than that associated with Erdafitinib. Conversely, EV demonstrates a significantly lower reporting rate for eye disorders, injuries, poisonings, procedural complications, and for benign, malignant, and unspecified tumors which compared to Erdafitinib.

**TABLE 3:** ADR number and report rate of 20 SOCs of Enfortumab vedotin and Erdafitinib.

System organ classes	Enfortumab vedotin (N = 4793)	Erdafitinib (N = 2,149)
Blood and lymphatic system disorders	227 (4.70%)	13 (0.60%)
Cardiac disorders	51 (1.06%)	11 (0.51%)
Congenital familial and genetic disorders	1 (0.02%)	0 (0.00%)
Ear and labyrinth disorders	3 (0.06%)	3 (0.14%)
Endocrine disorders	8 (0.17%)	3 (0.14%)
Eye disorders	108 (2.25%)	198 (9.21%)
Gastrointestinal disorders	373 (7.78%)	219 (10.19%)
General disorders and administration site conditions	682 (14.23%)	545 (25.36%)
Hepatobiliary disorders	76 (1.59%)	22 (1.02%)
Immune system disorders	18 (0.38%)	3 (0.14%)
Infections and infestations	252 (5.26%)	78 (3.63%)
Injury poisoning and procedural complications	240 (5.01%)	157 (7.31%)
Investigations	224 (4.67%)	104 (4.84%)
Metabolism and nutrition disorders	310 (6.47%)	121 (5.63%)
Musculoskeletal and connective tissue disorders	79 (1.65%)	48 (2.23%)
Neoplasms benign malignant and unspecified incl cysts and polyps	183 (3.82%)	118 (5.49%)
Nervous system disorders	533 (11.12%)	86 (4.00%)
Product issues	10 (0.21%)	9 (0.42%)
Psychiatric disorders	51 (1.06%)	17 (0.79%)
Renal and urinary disorders	137 (2.86%)	26 (1.21%)
Reproductive system and breast disorders	11 (0.23%)	5 (0.23%)
Respiratory thoracic and mediastinal disorders	147 (3.07%)	53 (2.47%)
Skin and subcutaneous tissue disorders	992 (20.70%)	235 (10.94%)
Social circumstances	10 (0.21%)	2 (0.09%)
Surgical and medical procedures	11 (0.23%)	53 (2.47%)
Vascular disorders	56 (1.17%)	20 (0.93%)

The top five reported AEs for EV are as follows: skin and subcutaneous tissue disorders (20.70%), general disorders and administration site conditions (14.23%), nervous system disorders (11.12%), gastrointestinal disorders (7.78%), and metabolism and nutrition disorders (6.47%). In comparison, the top five AEs for Erdafitinib are: general disorders and administration site conditions (25.36%), skin and subcutaneous tissue disorders (10.94%), gastrointestinal disorders (10.19%), eye disorders (9.21%), and injury poisoning and procedural complications (7.31%). Among these SOCs, ADRs with a reporting rate exceeding 10% include three SOCs for EV and three SOCs for Erdafitinib.

### Most common ADRs for EV and Erdafitinib

Table 4 presents the 20 most frequently reported ADRs for EV and Erdafitinib, categorized as PTs within each SOC. Common ADRs for both drugs include fatigue, diarrhea, anorexia, and alopecia. EV exhibits a notably higher ADR reporting rate for neuropathy peripheral, rash and fatigue. In contrast, Erdafitinib shows a greater incidence of for diarrhoea, and hyperphosphatemia. It is noteworthy that the mortality rate for Erdafitinib (8.34%) is significantly higher than that for EV (1.22%).

**TABLE 4:** Top 20 ADRs of Enfortumab vedotin and Erdafitinib.

Enfortumab vedotin (N = 6,205)		Erdafitinib (N = 2,758)	
ADR	Report rate %	ADR	Report rate %
Neuropathy peripheral	5.12	Death	8.34
Rash	5.12	Diarrhoea	3.37
Fatigue	2.69	Off label use	3.30
Malignant neoplasm progression	2.40	Hyperphosphataemia	2.57
Diarrhoea	2.26	Disease progression	2.28
Pruritus	2.00	Transitional cell carcinoma	1.92
Asthenia	1.79	Mucosal inflammation	1.89
Hyperglycaemia	1.68	Stomatitis	1.81
Nausea	1.60	Dry mouth	1.74
Skin toxicity	1.37	Onycholysis	1.67
Off label use	1.35	Fatigue	1.52
Decreased appetite	1.35	Nail disorder	1.49
Alopecia	1.29	Dry eye	1.41
Stevens-johnson syndrome	1.24	Alopecia	1.34
Death	1.22	Nail toxicity	1.34
Neutropenia	1.14	Palmar-plantar erythrodysaesthesia syndrome	1.23
Acute kidney injury	0.98	Decreased appetite	1.16
Product use issue	0.95	Drug ineffective	1.12
Erythema	0.95	Blood phosphorus increased	1.12
Weight decreased	0.90	Dry skin	1.12

Although the top 20 commonly reported AEs are mostly self-limiting and mild, notable events include malignant tumor progression associated with EV and disease progression linked to Erdafitinib.

### Serious AEs of EV and Erdafitinib

**FIGURE 1:**
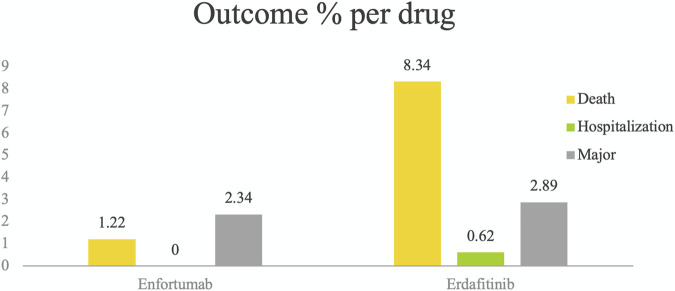
Serious adverse events identified for EV and Erdafitinib using WHO-VigiAccess, including life-threatening events, hospitalization, and disease progression. The proportion of reports indicating serious adverse reactions is 3.56% for EV and 11.85% for Erdafitinib.

Using WHO-VigiAccess, we can identify significant AEs for EV and Erdafitinib, including life-threatening events, hospitalization, and disease progression. The proportion of reports indicating serious adverse reactions is 3.56% for EV and 11.85% for Erdafitinib (Figure 1).

### Similarities and differences in common ADRs of EV and Erdafitinib

A comparison of ADRs reported for EV and Erdafitinib across SOCs identified 109 shared signals, as detailed in Table 5. The SOCs with the highest number of adverse signals are gastrointestinal disorders and general disorders including administration site conditions. For gastrointestinal disorders, the five most commonly reported reactions are nausea, gastrointestinal disorder, dry mouth, abdominal pain, and diarrhea. For general disorders and administration site conditions, the primary reported reactions include condition aggravated, feeling abnormal, mucosal inflammation, pain and disease progression.

**TABLE 5:** Same ADRs among Enfortumab vedotin and Erdafitinib.

System organ classes	ADRs	Signal N
Blood and lymphatic system disorders	Thrombocytopenia and anaemia	2
Cardiac disorders	Cardiac disorder	1
Eye disorders	Eye disorder, ocular toxicity, keratitis, visual impairment, vision blurred, lacrimation increased, and dry eye	7
Gastrointestinal disorders	Nausea, gastrointestinal disorder, dry mouth, abdominal pain upper, abdominal pain, diarrhoea, vomiting, dysphagia, constipation, colitis, and stomatitis	11
General disorders and administration site conditions	Condition aggravated, feeling abnormal, mucosal inflammation, pain, disease progression, asthenia, fatigue, general physical health deterioration, drug ineffective, death, and malaise	11
Hepatobiliary disorders	Liver disorder, hepatitis, cholestasis, hepatotoxicity, and hepatic failure	5
Immune system disorders	Hypersensitivity	1
Infections and infestations	COVID-19, infection, nasopharyngitis, staphylococcal infection, conjunctivitis, pneumonia, herpes zoster, urinary tract infection, and sepsis	9
Injury, poisoning and procedural complications	Product use in unapproved indication, off label use, toxicity to various agents, fall, incorrect dose administered, product use issue, and inappropriate schedule of product administration	7
Investigations	Blood alkaline phosphatase increased, weight decreased, alanine aminotransferase increased, blood creatinine increased, aspartate aminotransferase increased, transaminases increased, liver function test increased, blood bilirubin increased, hepatic enzyme increased, and gamma-glutamyltransferase increased	10
Metabolism and nutrition disorders	Dehydration, hyponatraemia, decreased appetite, hypercalcaemia, hypophosphataemia, and electrolyte imbalance	6
Musculoskeletal and connective tissue disorders	Arthralgia, myalgia,and pain in extremity	3
Neoplasms benign, malignant and unspecified (incl cysts and polyps)	Malignant neoplasm progression, neoplasm progression, metastases to liver, and bladder cancer	4
Nervous system disorders	Somnolence, dizziness, neuropathy peripheral, burning sensation, paraesthesia, taste disorder, dysgeusia, balance disorder, headache, and ageusia	10
Psychiatric disorders	Insomnia and confusional state	2
Renal and urinary disorders	Renal failure, renal impairment, and acute kidney injury	3
Respiratory, thoracic and mediastinal disorders	Interstitial lung disease, oropharyngeal pain, respiratory distress, pneumonitis, cough, dyspnoea, pulmonary embolism, hypoxia, and dysphonia	9
Skin and subcutaneous tissue disorders	Blister, pruritus, erythema, dry skin, alopecia, skin exfoliation, skin toxicity, and rash	8
Vascular disorders	Hypotension	1

When examining the top 20 ADRs reported for EV and Erdafitinib across SOCs (Table 6), distinct PTs emerge for each drug. EV is associated with a greater variety of unique symptoms in hematologic and lymphatic system disorders, endocrine disorders, and metabolic and nutritional diseases. In contrast, Erdafitinib exhibits more unique ADRs in eye disorders, tumors (benign, malignant, and unspecified), as well as surgical and medical procedures.

**TABLE 6:** Different ADRs among Enfortumab vedotin and Erdafitinib.

System organ classes	Enfortumab vedotin	Erdafitinib
Blood and lymphatic system disorders	Eosinophilia, lymphopenia, febrile neutropenia, pancytopenia, febrile bone marrow aplasia, bone marrow failure, haematotoxicity, lymphadenopathy, neutropenia, cytopenia, and leukopenia	Monocytosis
Cardiac disorders	Myocardial infarction, atrial fibrillation, and cardiac arrest	Atrioventricular block second degree
Endocrine disorders	Hypothyroidism	Thyroid disorder
Eye disorders	Eye irritation, eye pruritus, and blepharitis	Corneal thinning, retinopathy, eye swelling, maculopathy, central serous chorioretinopathy, chorioretinopathy, retinal oedema, retinal detachment, ulcerative keratitis, detachment of retinal pigment epithelium, serous retinal detachment, cataract, and blindness
Gastrointestinal disorders	Abdominal discomfort, small intestinal obstruction, and pancreatitis	Oral pain, rectal haemorrhage, lip pain, oral discomfort, chapped lips, mouth ulceration, intestinal obstruction, aphthous ulcer, and ascites
General disorders and administration site conditions	Therapy partial responder, oedema peripheral, extravasation, multiple organ dysfunction syndrome, gait disturbance, administration site extravasation, pyrexia, chills, and infusion site extravasation	Xerosis, adverse event, discomfort, mucosal disorder, drug intolerance, illness, adverse drug reaction, therapeutic product effect decreased, and adverse reaction
Hepatobiliary disorders	Hepatic cytolysis, hyperbilirubinaemia hypertransaminasaemia, and hepatic function abnormal	Drug-induced liver injury and cholangitis
Infections and infestations	Oral candidiasis, erysipelas, cellulitis, pneumonia aspiration, device related infection, clostridium difficile colitis, escherichia urinary tract infection, septic shock, and urosepsis	Paronychia, nail infection, nail bed infection, oral fungal infection, onychomycosis, haematological infection, and retinitis
Injury, poisoning and procedural complications	Infusion related reaction, underdose, and intercepted product storage error	Product use complaint, wrong technique in product usage process, intentional product use issue, medication error, product prescribing error, product dose omission issue, fracture, and product dispensing error
Investigations	Blood glucose abnormal, haemoglobin decreased, heart rate increased, white blood cell count increased, blood glucose increased, neutrophil count decreased	Blood phosphorus increased, liver function test abnormal, platelet count decreased, blood sodium decreased, lymphocyte count decreased, general physical condition abnormal, blood urine present, and white blood cell count decreased
Metabolism and nutrition disorders	Insulin resistance, hyperglycaemia, ketoacidosis, hypomagnesaemia, diabetic metabolic decompensation, hypophagia, hypokalaemia, diabetes mellitus, hypocalcaemia, diabetic ketoacidosis, and diabetes mellitus inadequate control	Hypoalbuminaemia, hyperphosphataemia, feeding disorder, and calciphylaxis
Musculoskeletal and connective tissue disorders	Back pain, mobility decreased, and muscular weakness	Muscle spasms, growth accelerated, tendon disorder, and bone pain
Neoplasms benign, malignant and unspecified (incl cysts and polyps)	Metastases to lung, and metastases to bone	Metastases to central nervous system, transitional cell carcinoma, metastatic carcinoma of the bladder, neoplasm, metastatic neoplasm, neoplasm malignant, brain neoplasm malignant, and malignant neoplasm of renal pelvis
Nervous system disorders	Hypoaesthesia, chronic inflammatory demyelinating polyradiculoneuropathy, peripheral sensory neuropathy, polyneuropathy, neuralgia, peripheral sensorimotor neuropathy, neurotoxicity, encephalopathy, peripheral motor neuropathy, and cognitive disorder	Central nervous system lesion, hyperaesthesia, syncope, spinal cord compression, cerebrovascular accident, mental impairment, and myelopathy
Product issues	Product temperature excursion issue	Product availability issue
Psychiatric disorders	Delirium	Eating disorder
Renal and urinary disorders	Haematuria, and tubulointerstitial nephritis	Urinary retention
Respiratory, thoracic and mediastinal disorders	Immune-mediated lung disease, dyspnoea exertional, respiratory failure, lung opacity, aspiration, lung disorder, and acute respiratory failure	Throat tightness, nasal dryness, haemoptysis, dry throat, pleural effusion, pulmonary oedema, epistaxis, and oropharyngeal discomfort
Skin and subcutaneous tissue disorders	Stevens-johnson syndrome, skin lesion, rash maculo-papular, rash vesicular, toxic skin eruption, skin reaction, toxic epidermal necrolysis, dermatitis bullous, symmetrical drug-related intertriginous and flexural exanthema, rash erythematous, rash pruritic, and skin discolouration	Onychomadesis, skin disorder, nail dystrophy, nail toxicity, nail discomfort, skin fissures, nail disorder, nail discolouration, palmar-plantar erythrodysaesthesia syndrome, onychoclasis, onychalgia, and onycholysis
Surgical and medical procedures		Therapy cessation, spinal operation, hospice care, surgery, therapy change, therapy interrupted, and hospitalisation
Vascular disorders	Hypertension, and shock	Deep vein thrombosis, embolism, dry gangrene, and thrombosis

Notably, EV is linked to more severe ADRs in skin and subcutaneous tissue disorders, including Stevens-Johnson syndrome (SJS) and toxic epidermal necrolysis (TEN). Conversely, Erdafitinib demonstrates a broader spectrum of ADRs in eye disorders, encompassing corneal thinning, retinal disorders, and macular degeneration.

## Discussion

This study elucidates the safety profiles of EV and Erdafitinib, two novel drugs for the treatment of bladder cancer, through an analysis of post-market ADR reports from the WHO-VigiAccess database.

Geographically, the majority of ADR reports originate from the Americas and Europe, consistent with previous studies on ADR associated with other drugs. For instance, Varallo and Forgerini noted that developed countries typically possess more advanced pharmacovigilance systems, resulting in a higher volume of ADR reports (Varallo et al., 2019). Conversely, the lower reporting rates from Asia, Africa, and Oceania may indirectly reflect inadequacies in pharmacovigilance systems and reporting mechanisms in these regions, or potentially a lower usage of these drugs. Kiguba et al. emphasized the challenges faced by developing countries in establishing effective pharmacovigilance systems, including resource constraints and a lack of trained personnel (Kiguba et al., 2023). This finding underscores the necessity of strengthening global pharmacovigilance systems, particularly in developing countries.

The analysis of gender distribution indicates that EV and Erdafitinib exhibit a higher reporting rate in males. Smoking is a well-established risk factor for bladder cancer (Strope and Montie, 2008). Studies suggest that women may be more susceptible to bladder cancer than men (Scosyrev et al., 2009). Interestingly, the incidence of bladder cancer is approximately three to four times higher in men than in women (Krabbe et al., 2015; Dobruch et al., 2016). Several studies have explored the potential molecular mechanisms underlying this disparity. Zhang proposes that this difference may be linked to variations in liver metabolism and the detoxification of carcinogens. Specifically, uridine 5′-diphospho-glucuronosyltransferase (UGT), which is involved in the metabolism of aromatic amines, can reduce the harmful components found in tobacco smoke, while androgen receptor (AR) signaling may suppress this detoxification pathway (Zhang, 2013). Additionally, microbial differences in the urinary tract have been implicated in this gender disparity. *Lactobacillus* predominating in women, whereas Corynebacterium is more common in men (Xu et al., 2014). A previous clinical trial demonstrated a protective effect of oral *Lactobacillus* supplementation against bladder cancer recurrence, however further clinical data are required to substantiate this finding (Aso et al., 1995). Other studies suggest that the combination of estrogen and progesterone may reduce the risk of bladder cancer (McGrath et al., 2006; Davis-Dao et al., 2011; Cantwell et al., 2006). Jubber et al. reported that this variability may be related to drug use patterns, but further research and discussion are needed (Jubber et al., 2023). In terms of age distribution, EV and Erdafitinib show a higher reporting rate among elderly patients aged 65–74 years. As physiological functions gradually decline with age, the likelihood of elderly individuals developing various complications increases. This decline also impacts drug metabolism within the body, significantly elevating the risk of AEs (Budnitz et al., 2011; Budnitz et al., 2006).

There are notable differences in the common types of ADRs associated with the two medications. EV is primarily linked to skin and subcutaneous tissue disorders and nervous system disorders, while Erdafitinib is chiefly associated with eye disorders, metabolic disturbances and skin and subcutaneous tissue disorders. These differences may arise from the distinct mechanisms of action of the two drugs.

EV has demonstrated remarkable efficacy in the treatment of blaader cancer. Due to its mechanism of targeting nectin-4, EV is also being investigated for the treatment of other nectin-4-expressing malignancies, including gastrointestinal tumors, small cell lung cancer, and breast cancer (Chatterjee et al., 2021; Challita-Eid et al., 2016). EV targets nectin-4 to deliver the cytotoxic agent MMAE into cancer cells, where MMAE disrupts microtubule polymerization in keratinocytes, inducing apoptosis or necrosis and achieving a tumor-specific antitumor effect. In the EV-201 trial, Rosenberg et al. reported skin toxicity and peripheral neuropathy as notable adverse effects of EV, with skin toxicity typically emerging within the first or second cycle (Rosenberg et al., 2023). Severe reactions, such as SJS and TEN, have also been observed (Nguyen et al., 2021). These findings underscore the necessity of closely monitoring skin reactions during EV therapy. The mechanisms underlying these adverse reactions may be associated with the physiological expression of nectin-4 in keratinocytes and sweat glands. Under normal skin conditions, the targeting of nectin-4 by EV could result in skin-related AEs. But in pathological skin conditions, EV may represent a potential therapeutic option. For instance, increased nectin-4 expression has been noted in a subset of cutaneous adnexal carcinomas, particularly sebaceous carcinomas, indicating that EV may be a promising treatment for these tumors (Ingen-Housz-Oro et al., 2024; Cho et al., 2024). Alternatively, it has been suggested that these adverse effects may be mediated by a Type IV hypersensitivity reaction. Actively dividing epidermal keratinocytes are particularly vulnerable to the anti-mitotic effects of MMAE (Doronina et al., 2003). Damaged keratinocytes release antigens that activate dendritic cells, leading to antigen presentation to T cells. These T cells subsequently activate other immune cells, such as effector T cells, and secrete cytokines and chemokines, including tumor necrosis factor-α (TNF-α) and interleukin-2 (IL-2). The release of cytokines and chemokines triggers an inflammatory response and tissue damage, which manifests in the skin as erythema, edema, rash, and pain. In severe cases, this may progress to SJS or TEN (Phillips et al., 2019; Duong et al., 2017; Tan and Tan, 2011; Bellón, 2019). Additionally, the bystander effect induced by EV may provoke immune responses in adjacent healthy tissue, further exacerbating the damage (Liu et al., 2020). Furthermore, Ingen-Housz-Oro et al. provided detailed insights into EV-related skin toxicity in the EV-301 trial, proposing various management strategies to mitigate these adverse effects (Ingen-Housz-Oro et al., 2024). Peripheral neuropathy, in contrast, tends to appear later in treatment cycles (Zschäbitz et al., 2023). Research by Taoka et al. demonstrated that EV significantly impacts sensory nerves, especially in the lower limbs, with the sural nerve being particularly susceptible (Taoka et al., 2024). This neurotoxicity may correlate with the amount of MMAE released following EV uptake by cancer cells, suggesting a positive relationship between therapeutic efficacy and the incidence of peripheral neuropathy.

In comparison, the BLC2001 study conducted by Zheng et al. identified ocular toxicity and metabolic disturbances associated with Erdafitinib (Zheng et al., 2022). Ocular adverse reactions linked to Erdafitinib, including corneal thinning, retinal disorders, and macular degeneration, likely a consequence of its FGFR inhibitory action, as FGFRs play a critical role in the cell growth and maintenance of retinal cells. The inhibition of FGFRs may disrupt normal retinal and corneal functions, potentially leading to retinal detachment and changes in the corneal epithelium (Ouwerkerk and Boers-Doets, 2010). One study suggests that this inhibition can block the activation of the mitogen-activated protein kinase (MAPK/MEK) signaling cascade, resulting in MEK-related retinal disorders (Sassine et al., 2024). This finding aligns with the safety profiles observed for other FGFR inhibitors and underscores the importance of conducting routine ophthalmologic assessments during Erdafitinib therapy (Borkar et al., 2013). Hsu et al. have proposed management strategies to address FGFR inhibitor-induced ocular toxicity, which may help mitigate the ocular adverse effects associated with Erdafitinib treatment (Hsu et al., 2024). Concerning metabolic disturbances, the underlying machanism is primarily linked to FGFR signaling in phosphate metabolism. FGFR1, which plays a critical role in the skeletal system, regulates the secretion of fibroblast growth factor 23 (FGF23), a hormone produced by osteocytes that reduces serum phosphate levels by decreasing renal phosphate reabsorption and lowering 1,25-dihydroxyvitamin D synthesis. By inhibiting FGFR1, Erdafitinib reduces FGF23 levels, which diminishes the inhibition of sodium-phosphate co-transporters in renal proximal tubules, thereby increasing renal phosphate reabsorption and resulting in hyperphosphatemia (Kommalapati et al., 2021). Additionally, FGFR inhibitors may impact other metabolic pathways. For example, FGFRs are involved in insulin signaling, and their inhibition could lead to insulin resistance, affecting blood glucose levels (Wöhrle et al., 2011). However, further research is required to clarify the specific mechanisms of other metabolic abnormalities. In clinical practice, monitoring serum phosphate levels during Erdafitinib therapy is essential. For patients experiencing hyperphosphatemia, dose adjustments or other interventions may be necessary to manage metabolic disturbances, ensuring both the safety and the efficacy of the treatment. Moreover, although both EV and Erdafitinib are associated with notable skin adverse reactions, their mechanisms are differ. FGFR is expressed in skin cells and various other tissues, playing a crucial role in cell proliferation, differentiation, and repair. Erdafitinib may induce dermatological adverse effects by inhibiting FGFR, which disrupts the normal growth and repair processes of skin cells. Similar dermatologic events are also observed with other targeted anticancer therapies and immunotherapies (Lacouture et al., 2021; Schneider et al., 2021; Lacouture and Sibaud, 2018).

In our study, it is noteworthy that the number of ADR reports associated with EV is significantly higher than that for Erdafitinib, yet the mortality rate linked to EV is markedly lower (EV 1.22%, Erdafitinib 8.34%). This discrepancy may be attributed to the distinct nature of the adverse reactions associated with each drug. The primary side effects of EV include skin reactions and peripheral neuropathy, which can be severe but are not necessarily fatal. In contrast, the adverse effects of Erdafitinib, such as ocular disorders and metabolic abnormalities like hyperphosphatemia, may lead to more serious complications that could impact patient survival. Additionally, our study indicate there is no significant difference between the two drugs in terms of hospitalization, the possible reason is the side effects of both drugs can often be managed through outpatient care or short-term hospitalization.

While this study offers valuable insights, it is not without limitations. Firstly, SRS data may be subject to reporting biases, including notoriety bias and selective reporting. Hauben and Aronson discussed the implications of these biases for the interpretation of pharmacovigilance data (Hauben and Aronson, 2009). Secondly, the lack of accurate exposure population information renders the calculation of true ADRs incidence rates unfeasible. Bate and Evans highlighted this limitation and proposed potential remedies (Bate and Evans, 2009). Furthermore, as the WHO-VigiAccess database comprises cumulative data, it lacks annual ADRs data, which restricts the analysis of ADR trends.

This study provides valuable insights into the post-marketing safety of EV and Erdafitinib through an analysis of the WHO-VigiAccess database. The results underscore the distinct safety profiles of these two drugs, providing crucial information for clinical practice. However, given the inherent limitations of SRS data, these findings should be interpreted in conjunction with evidence from other sources. Based on these considerations, we propose several potential directions for future research as useful references. First of all, global pharmacovigilance systems need to be strengthened. Despite the ongoing introduction of new drugs, pharmacovigilance systems in various countries continue to face significant challenges. To more comprehensively assess the post-marketing safety of drugs, enhancements to global pharmacovigilance systems are essential particularly in developing countries. Improvements in drug monitoring systems in these regions will enhance the efficiency and accuracy of safety monitoring (Conn and Ruppar, 2017). Furthermore, stricter drug safety monitoring is necessary: big data and artificial intelligence technologies can be employed to analyze large datasets from multiple sources, including clinical trials, electronic health records, and patient feedback, thereby improving the accuracy and efficiency of drug safety monitoring (Ajlan et al., 2019). Patient adherence is a key factor influencing the incidence of ADRs, and there exists a complex interplay between the two (Conn and Ruppar, 2017). Studies indicate that the average adherence rate in developed countries is approximately 50% (Brown and Bussell, 2011). Patients with poor adherence are more likely to experience or exacerbate ADRs, while the occurrence of ADRs further diminishes adherence (Ho et al., 2009). Therefore, improving patient adherence not only enhances therapeutic efficacy but also reduces ADRs and complications, lowers healthcare costs, and improves the overall quality of life for patients. Current strategies for improving patient adherence primarily focus on the patient level and include individualized treatment, supervision and management, patient education, medication reminder systems, behavioral incentives, adherence assessment tools, and psychological interventions (Morisky et al., 1986; Madhombiro et al., 2019). Among these strategies, individualized treatment and supervision are crucial. Future drug therapies will increasingly be personalized, with treatment plans tailored to the genetic of patients, environmental, and health status. Research indicates that EV is more suitable for patients with healthy skin and neurological systems, a robust immune status, and no significant comorbidities, while Erdafitinib is best suited for patients with FGFR gene mutations, no severe ocular or metabolic diseases, and those willing to undergo regular monitoring. Additionally, strengthening supervision and management is key to preventing ADRs and reducing associated risks. For EV, it is recommended to regularly assess skin and neurological health, alongside the preventive use of zinc-based moisturizers to protect the skin from UV-induced damage. In cases of severe ADRs (such as SJS or TEN), treatment should be discontinued immediately, and patients should be transferred to intensive care for supportive therapy (Yu et al., 2020; Salzmann et al., 2019; Chen et al., 2018). For Erdafitinib, patients should undergo baseline ocular and metabolic screening prior to treatment and be monitored regularly throughout therapy, particularly in elderly patients. In cases of severe retinal disease, hyperphosphatemia, or other life-threatening ADRs, treatment should be discontinued, and appropriate interventions initiated. In conclusion, a patient-centered approach involving enhanced pre-treatment education, individualized treatment plans, strengthened monitoring during therapy, and collaborative rehabilitation post-treatment can effectively improve patient adherence, reduce ADR incidence, maximize therapeutic efficacy, and ultimately improve patient outcomes.

Developing new drugs or repurposing existing ones offers great potential. Current treatments for bladder cancer primarily consist of immunotherapy and targeted therapies, often used in combination to achieve better outcomes (Bader et al., 2020; Meric-Bernstam et al., 2021). However, these methods carry safety concerns due to adverse reactions (Galon and Bruni, 2019). Future research should validate the efficacy and safety of combination therapies through larger clinical trials. Targeting specific tumor molecular drivers also represents a promising avenue for exploration (Shariati and Meric-Bernstam, 2019; Li D. X. et al., 2023; Shen et al., 2024). Moreover, exploring the molecular mechanisms of ADRs is vital. Understanding drug interactions with immune systems, metabolic pathways, or cytotoxic responses, and validating findings through cohort studies or randomized trials, is crucial for identifying the causes of side effects. This will pave the way for improved prevention and treatment strategies.

## Conclusion

EV and Erdafitinib are essential drugs in the treatment of bladder cancer, making their safety and toxicity management crucial. According to data from the WHO-VigiAccess database, these two drugs have a substantial number of ADR reports, with EV reporting 2,257 cases and Erdafitinib reporting 1,181 cases. The ADRs primarily involve skin and subcutaneous tissue disorders, general disorders, nervous system disorders, and gastrointestinal issues. Specifically, EV is more likely to cause skin and nervous system-related adverse reactions, whereas Erdafitinib is closely associated with eye disorders and metabolic disturbances. Notably, the mortality rate for Erdafitinib is 8.34%, significantly higher than the 1.22% associated with EV. Although most ADRs are relatively mild, some severe reactions may necessitate hospitalization or even result in death. Therefore, countries should actively conduct safety studies on biologic agents, with a particular focus on monitoring ADRs in real-world applications to better understand the risk-benefit profile of these therapies. Establishing robust monitoring systems and data collection mechanisms, especially for analyzing impacts across diverse populations and patients with specific diseases, is essential for elucidating the causal relationship between ADRs and individual drugs. Furthermore, personalized strategies should be developed based on ADR characteristics; for instance, EV may be more appropriate for patients at risk of ocular issues or metabolic disturbances, whereas Erdafitinib might be a more suitable choice for those with potential risks related to skin or nervous system complications. This approach aims to reduce ADR risk in specific patient populations, enhance overall therapeutic outcomes, and provide more effective, targeted treatment options for bladder cancer patients.

## Data Availability

The original contributions presented in the study are included in the article/supplementary material, further inquiries can be directed to the corresponding authors.
